# High-Frequency Electroencephalographic Activity in Left Temporal Area Is Associated with Pleasant Emotion Induced by Video Clips

**DOI:** 10.1155/2015/762769

**Published:** 2015-03-26

**Authors:** Jukka Kortelainen, Eero Väyrynen, Tapio Seppänen

**Affiliations:** Department of Computer Science and Engineering, University of Oulu, P.O. Box 4500, 90014 Oulu, Finland

## Abstract

Recent findings suggest that specific neural correlates for the key elements of basic emotions do exist and can be identified by neuroimaging techniques. In this paper, electroencephalogram (EEG) is used to explore the markers for video-induced emotions. The problem is approached from a classifier perspective: the features that perform best in classifying person's valence and arousal while watching video clips with audiovisual emotional content are searched from a large feature set constructed from the EEG spectral powers of single channels as well as power differences between specific channel pairs. The feature selection is carried out using a sequential forward floating search method and is done separately for the classification of valence and arousal, both derived from the emotional keyword that the subject had chosen after seeing the clips. The proposed classifier-based approach reveals a clear association between the increased high-frequency (15–32 Hz) activity in the left temporal area and the clips described as “pleasant” in the valence and “medium arousal” in the arousal scale. These clips represent the emotional keywords amusement and joy/happiness. The finding suggests the occurrence of a specific neural activation during video-induced pleasant emotion and the possibility to detect this from the left temporal area using EEG.

## 1. Introduction

The understanding and measurement of emotional experiences is a critical task in affective computing, a nascent field of study to understand the technological implications and possibilities of emotional computing [1]. After a few centuries of scientific study, the current understanding of emotional expressions and the multimodal nature of audiovisual experience of emotion have evolved much from the early treatises on emotion [2]. Old views that attribute emotions and expressions thereof to monopolar emotional labels of acquired qualities or even God-given abilities [2], which were famously countered by Darwin in his classic book [3] have been superseded by modern approaches. The search for an atomic fundamental representation of affect, beyond the concept of basic emotions popular during the last half century [4], has resulted in, among other models of emotion, for example, component models of cognitive appraisal, the modern paradigm of dimensional model of emotion in the last few decades [5–7]. While static emotional labels are still very much relevant, a two-dimensional bipolar circumplex model of valence and arousal [5] can be seen as an essential representation of the affective space enhancing the strict label-based views of categorical emotions by integrating the emotional labels into a looser more malleable continuous structure. The dimensional model of valence and arousal is thus an important foundation for the technological study of emotion allowing, for example, via a projection of distinct emotional class labels into a low dimensional representation, a more efficient description of emotional data.

Due to the recent advance in functional imaging modalities, certain brain areas, such as limbic system's anterior cingulate cortex, amygdala, orbitofrontal cortex, and insular cortex, have been associated with the processing of emotional stimuli [8]. Studies have also been addressed to explore the distinct brain systems responsible for the processing of valence and arousal [9–11]. The findings suggest that specific neural correlates for these key elements of basic emotions do exist and can be identified by neuroimaging techniques [12].

Electroencephalogram (EEG) represents one of the modalities frequently applied for emotion recognition in recent studies. Compared to other imaging techniques, such as the functional magnetic resonance imaging, EEG provides certain obvious advantages. In addition to its high temporal resolution, the recordings can be carried out with low-priced and portable equipment. The novel easy-to-attach and even wireless measurement systems have made the usage of EEG possible also outside the clinical environment. The EEG-based technological solutions for emotion recognition have conventionally relied on assessing the activity changes in the classical frequencies, that is, delta (1–4 Hz), theta (5–8 Hz), alpha (9–12 Hz), beta (13–30), and gamma (>30 Hz) bands, as well as the activity differences between the hemispheres. The electrical activity of the brain was shown to be affected by emotions more than 30 years ago [13] after which an extensive amount of research related to EEG-based emotion recognition has been carried out. For example, approaches for classifying music- [14] and picture-induced [15] emotions have been proposed. Emotion classification using EEG after audiovisual stimulation has also been widely studied. Murugappan et al. successfully carried this out using discrete wavelet transform with fuzzy *c*-means and fuzzy *k*-means clustering [16] and later with *k* nearest neighbors and linear discriminant analysis [17]. Although the role of frontal EEG activity in video-induced emotion recognition has been emphasized [18], lately novel approaches such as functional connectivity pattern analysis of different topographic areas of the brain have also been proposed [19]. While still being in its infancy, emotion recognition using EEG can be seen to provide a huge scientific potential from both neuroscience and technological points of view.

In this paper, the EEG markers for video-induced emotions are explored. Even though the literature presents several approaches for this (see, e.g., [19, 20]), no compelling evidence of markers consistently seen across different studies is provided. One of the reasons for this might be in the common practice of restricting the analysis to the classical frequency bands (delta, theta, alpha, beta, and gamma), even though narrower bands have been suggested to better reveal the emotion-related changes in EEG [21]. Marosi et al. found specific bands within alpha and beta ranges to be indicative of the person's emotional state and concluded that the usage of classical frequency bands may cause the frequency-specific effects to go undetected or cancel each other. The restriction is mainly to avoid the difficulties related to the analysis of the huge amount of data produced by higher number of narrower bands. Current paper overcomes this problem by a novel classifier-based approach: the features that perform best in classifying the emotion of subjects watching video clips with emotional content are selected from a large feature set generated using a high number of partly overlapping frequency bands of varying width. The feature selection is carried out by applying a sequential forward floating search method to the feature set reduced in advance by a statistical preselection. The selection is performed to construct two different feature sets optimizing the classification of valence and arousal, both derived from the emotional keywords the subjects chose after seeing the clips. The features are comprised of the spectral powers (SPs) of single channels as well as the spectral power differences (SPDs) between specific channel pairs located symmetrically on the different hemispheres. Compared to the previous studies, this approach provides an exceptional possibility to observe empirically derived topographic and frequency characteristics, not restricted by the classical frequency bands of EEG, related to emotional experience during audiovisual stimulation. We hypothesize that there exists a topographic pattern of frequency-specific features that perform best in classification of person's emotional state while watching emotional video clips and that the features might not follow the classical frequency bands used in the EEG analysis. The findings are confirmed with a separate validation protocol in which also the contribution of electro-oculographic (EOG) and transient electromyographic (EMG) artifacts is assessed.

## 2. Materials and Methods

### 2.1. Data Collection

The study was carried out using the MAHNOB Database [22]. The database is available online (http://www.ibug.doc.ic.ac.uk/resources/mahnob-hci-tagging-database/) and contains recordings of user responses to multimedia content. The original experimental protocol consisted of two parts, from which only the first one was used in this study.

A detailed description of the experimental protocol and data collection is given in [22]. During the experiment, fragments of videos with emotional content were shown to 30 subjects (13 males and 17 females). In addition to visual content, the video clips contained music and speech. The participants were 19–40 years old (26.06 ± 4.39) and came from different cultural backgrounds. The handedness of the subjects was not controlled. The instructions for the experiment were given in English. The video material, consisting of 20 clips 34.9–117 s, in duration, was taken from commercially produced movies (14) or online resources (6). The clips, representative of different emotions, were selected based on the results of a preliminary study conducted utilizing an online affective video annotation system [22]. The 20 selected clips represented six different emotions: neutral (3), amusement (3), joy (5), disgust (3), fear (3), and sadness (3). The clips were played in a random order. After each clip, the participant was asked to annotate their emotive state using a keyword. The annotation was performed by pressing a numerical key. The keyword was chosen from nine possibilities: neutral, surprise, amusement, joy/happiness, disgust, anger, fear, sadness, and anxiety. Based on the division presented by Fontaine et al. [7], each keyword was then mapped into one of three classes according to the valence and arousal. The classes were “pleasant” (amusement, joy/happiness), “neutral valence” (neutral, surprise), and “unpleasant” (disgust, anger, fear, sadness, anxiety) for valence and “calm” (neutral, disgust, sadness), “medium arousal” (amusement, joy/happiness), and “excited/activated” (surprise, anger, fear, anxiety) for arousal. Each keyword-based annotation was thus translated to have a representative class in both valence and arousal scale. For examples, for keyword disgust the translation was “unpleasant” and “calm.”

While the subjects watched the videos, EEG was recorded using 32 active AgCl electrodes following the international 10/20 system of electrode placement. The recording was carried out with a sampling rate of 1024 Hz after which the signals were downsampled to 256 Hz. Common average reference was used. Due to unfinished data collection, technical problems, and signal artifact, only 541 of the 600 data recordings (20 clips for 30 subjects) could be included in the analysis. The data recordings (referred to as samples from this point on) left out included the whole data (20 samples) of two subjects. With the approach presented above, each EEG recoding could be associated with one of the three classes in both valence and arousal scales. Only the signal parts recorded while the participants watched the videos were included in the analysis, while the part recorded during the annotation was left out.

### 2.2. Feature Extraction

The EEG signal processing and data analysis presented in this paper have been carried out using the Matlab technical computing language (The MathWorks, Inc., Natick, MA).

For each EEG recoding, a power spectral density estimate was calculated using Welch's averaged periodogram method [23]. Hamming windowing with a window length of 5 s and overlap of 4 s was used. Features were then extracted from the estimates using the band 1–32 Hz. Firstly, the powers in all the single frequencies of the band were chosen as separate features. Secondly, the powers in all adjacent 2 Hz, 4 Hz, 8 Hz, and 16 Hz wide subbands were included in the feature set as well as the total power in the 1–32 Hz frequency band. These spectral power (SP) features were calculated for all 32 channels recorded. In addition, spectral power difference (SPD) features were determined by calculating the differences of the above described features between the 14 electrode pairs located symmetrically over the left and right hemispheres. The total number of features was thus 2898 including 2016 SP (63 frequency subbands × 32 channels) and 882 SPD (63 frequency subbands × 14 channel pairs) features. The above-described feature extraction approaches were shown to perform well with the used data in our previous work and including higher frequencies (>32 Hz) did not improve the result [24].

Due to several reasons, such as electrode impedance and anatomical differences, the absolute values of EEG may vary substantially between individuals. This variation was reduced by mapping the feature values separately for each subject into the range of [0, 1]. This feature normalization was carried out by subtracting the minimum value of the feature from all the feature values and then dividing the values by the difference between the maximum and minimum values of that specific feature. Similar feature normalization approach was used, for example, in [22].

### 2.3. Feature Selection

A sequential forward floating search method was applied to the original feature set aiming to reduce the dimensionality of the data and thereby improve the classification performance. The method was applied to the data separately for valence and arousal resulting in two different feature sets. The used method, proposed by Pudil et al. [25], is based on a sequential search of the best feature subset using dynamic inclusion and exclusion of features. In a nutshell, the algorithm comprises the following steps.

1. *Inclusion*. Inclusion of the features that are not yet in the feature set is tested one by one. The feature that leads to the best performance is included in the feature set.
2. *Conditional Exclusion*. Exclusion of the features that are already in the feature set is tested one by one. If there is a feature, whose removal leads to better performance compared to the performance received with the reduced feature set earlier, the feature is excluded from the feature set. If there is more than one such feature, the one whose removal leads to the best performance is selected.

At first, the feature set is empty. After performing step (1), step (2) is repeated until the criterion is not fulfilled. The algorithm then goes back to step (1). When all or a predefined number of features are included in the feature set, the algorithm terminates. While being computationally effective compared to, for example, the approach of trying all the feature combinations, the algorithm has been shown to provide an optimal or near optimal performance. The performance of the feature set was determined by the classification rate that was calculated using a *k* nearest neighbors leave-one-subject-out approach. Based on our previous studies with the same dataset, *k* = 3 was used in the classification [24].

As the number of features in the original feature set was high, the application of the feature selection algorithm to the whole set would have been computationally too demanding. Consequently, a preselection of features was carried out separately for valence and arousal using one-way ANOVA test. The test was performed separately for each feature in the whole data set with the class (i.e., “pleasant,” “neutral valence,” and “unpleasant” for valence) as the independent variable and the feature value as the dependent variable. Only the features for which a statistical threshold was exceeded (*P* < 0.2) were included in the further analysis, that is, the application of the above-described feature selection algorithm. The decision of the statistical threshold was based on the results of our previous work [24]. The chosen threshold represented the best compromise between reducing the number of features enough while not being restricted too strictly only to the features that linearly separate the classes.

### 2.4. Validation of the Results

In classification tasks, the usage of high number of features compared to the number of data samples may lead to overlearning. Usually, this problem is avoided by dividing the data into training and testing sets. However, as the amount of samples in the used dataset was rather small, leaving out a substantial part of the data for testing potentially deteriorates the findings. We therefore carried out a separate procedure for the validation of the result achieved with the whole dataset. In this procedure, six random samples were chosen from each participant to form the testing set. The above-described feature selection method including the preselection part was then applied to the rest of the data comprising the training set. By using an independent testing set, we were able to assess the classification performance and possible overlearning at different phases of feature selection and thereby validate the results achieved with the whole dataset.

Due to the experimental setup, some of the recordings were contaminated with complex EMG and EOG artifacts. Removal of these samples would have substantially reduced the amount of data. On the other hand, due to the partly overlapping spectral properties of the signals, the removal of the artifacts would have affected the EEG. Instead, an alternative approach was chosen. Automatic artifact detection was carried out to estimate the contribution of EMG and EOG to the data. With this approach, the role of the artifacts could be taken into account when interpreting the findings.

For EMG detection, the spectrogram for each sample was calculated using Short-time Fourier transform (1-s Hamming window, no overlap). The power of each 1-s signal segment was then determined in the frequency band > 70 Hz. If the power exceeded more than 10 times the median of that sample, the signal segment was classified to contain EMG. This approach detected reliably transient EMG artifacts assuming that less than half of the data sample was contaminated. The number of the segments containing EMG was then divided by the length of the sample to give a reference value for the contribution of EMG to that specific sample.

For the detection of EOG artifacts, the signal baseline was removed with median filtering. By applying a finite impulse response lowpass filter, the signal components higher than 20 Hz were then removed. The locations where the filtered signal exceeded a predefined threshold, that is, 2 times the standard deviation of the median filtered signal, were classified to contain EOG artifact. The number of EOG artifacts detected was divided by the length of the sample to give a reference value for the contribution of EOG to that specific sample.

### 2.5. Statistical Analysis

The feature values representing the activity in single frequencies between 1 and 32 Hz were statistically compared using one-way ANOVA test. The analysis was performed separately for valence and arousal using the class as the independent variable and the feature value as the dependent variable. *P* values less than 0.05 were considered to indicate statistical significance. The approach was used to find out clear differences in topographical as well as frequency space signal characteristics between groups. Due to the multiple comparisons, the statistical results were interpreted conservatively. For the most prominent finding related to the channel T7, the difference between all three pairs of classes was further explored with the Tukey post hoc honestly significant difference test. The amount of EMG and EOG artifact was also statistically compared between classes using a Mann-Whitney *U* test.

## 3. Results

**Figure 1:**
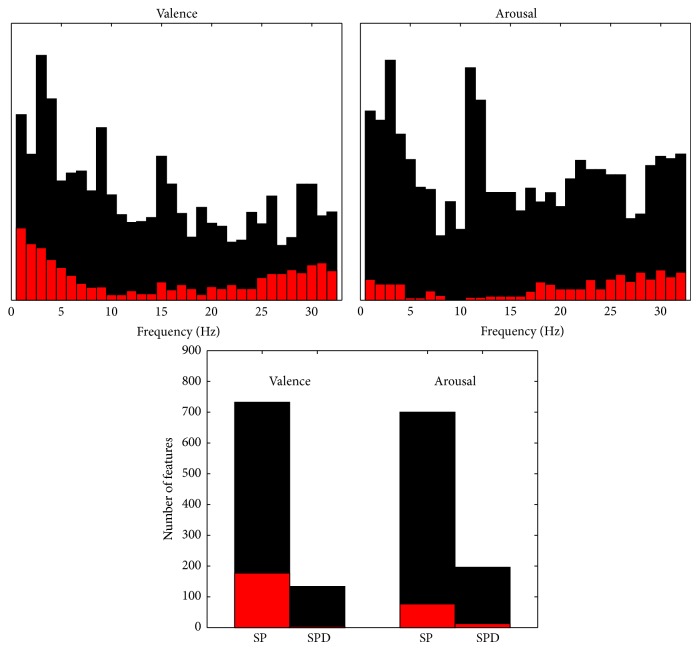
The contribution of different frequencies (upper row) and the number of features (lower row) in the preselected (black) and best performing (red) feature sets for valence and arousal. The contribution of different frequencies is illustrated in arbitrary scale so that each feature equally contributes to the area in the histogram. The features representing single frequencies increase the corresponding bins. The features representing more than one frequency, for example, 2 Hz wide bands, increase all the corresponding bins within the bands. The bins are, however, increased only by half compared to the previous case of single frequencies. The number of features is given separately for spectral power (SP) and spectral power difference (SPD) features.

After carrying out a statistical preselection for the features extracted, two separate feature sets were selected to optimize the EEG-based classification of person's valence and arousal while watching videos with emotional content. The contribution of different frequencies in the preselected feature set as well as the optimized (i.e., best performing) features set is illustrated in Figure 1 for both valence and arousal. The size of the preselected feature set was 866 features for valence and 896 for arousal while the number of features in the best performing feature set was 181 for valence and 90 for arousal. Generally, the contribution of low (<8 Hz) and high (>25 Hz) frequencies was emphasized in the classification of valence whereas, for arousal, the absence of features representing 5–15-Hz activity was notable. Figure 1 also shows that even though the preselected feature sets contained many SPD features, almost none of them were selected in the best performing feature set.

**Figure 2:**
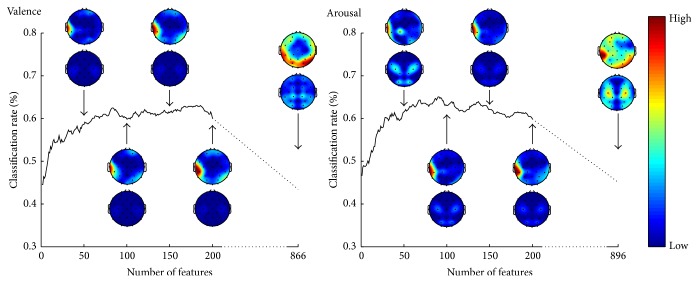
The classification performance and the topographical distribution of features as a function of number of features for valence and arousal. The topographic plots are given in relative scale red indicating high and blue low number of features. Spectral power (above) and spectral power difference (below) features are illustrated separately for 50, 100, 150, 200, and all preselected features.

The classification performance, that is, the percentage of correctly classified samples, in different phases of the feature selection is illustrated in Figure 2. The best classification rates were 63.0% and 65.1% for valence and arousal, respectively. The figure also shows how the features were topographically distributed over the scalp. The topographic plots are made using EEGLAB [26].

### 3.1. Valence

**Figure 3:**
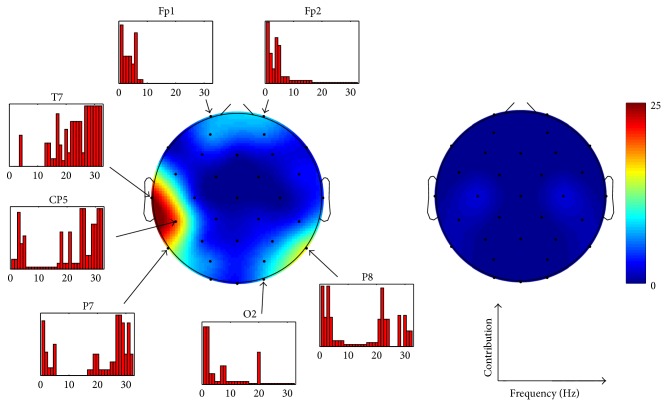
The topographical distribution of features and the channel-wise contribution of different frequencies in the best performing feature set for valence. The feature set contained 181 features. Spectral power (on the left) and spectral power difference (on the right) features are illustrated separately. The topographic plots are given in absolute scale representing the number of features.

**Figure 4:**
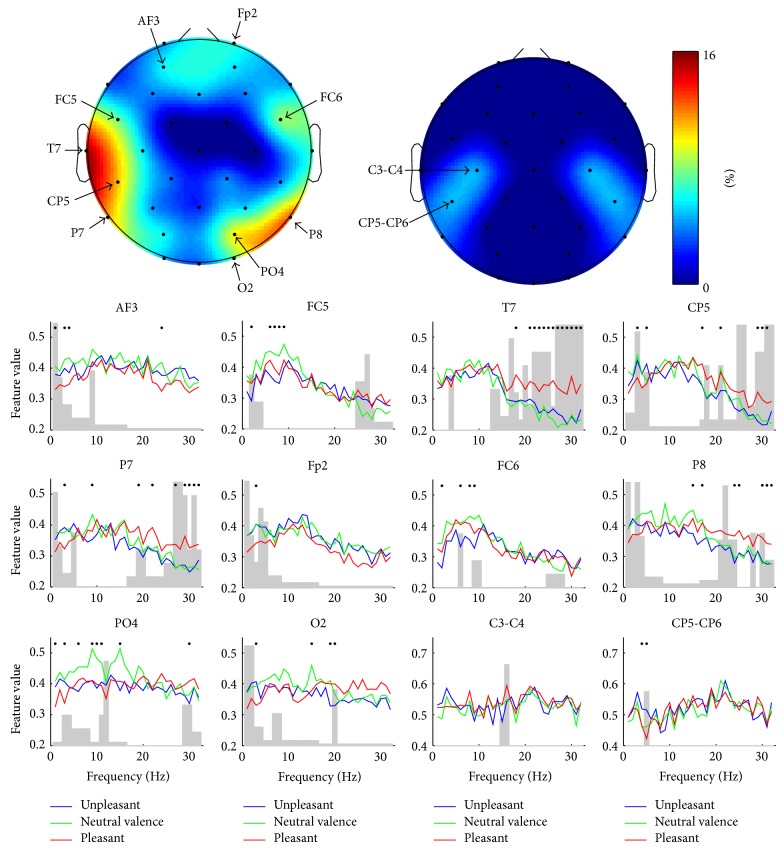
The importance of different channels and frequencies in the classification of valence. The topographic plots illustrate channel-wise how the classification performance is decreased if all the features in that specific channel are excluded from the best performing feature set. The plots have been made separately for the spectral power (on the left) and the spectral power difference (on the right) features. The channel-wise feature values for the three different classes of valence are given below the topographic plots. The curves are the mean values of the features representing the single frequencies. The dots above the curves indicate that the values between the classes in that specific frequency differ statistically significantly (*P* < 0.05) according to one-way ANOVA test. The gray bars represent the contribution of different frequencies in that specific channel in the best performing feature set.

**Figure 5:**
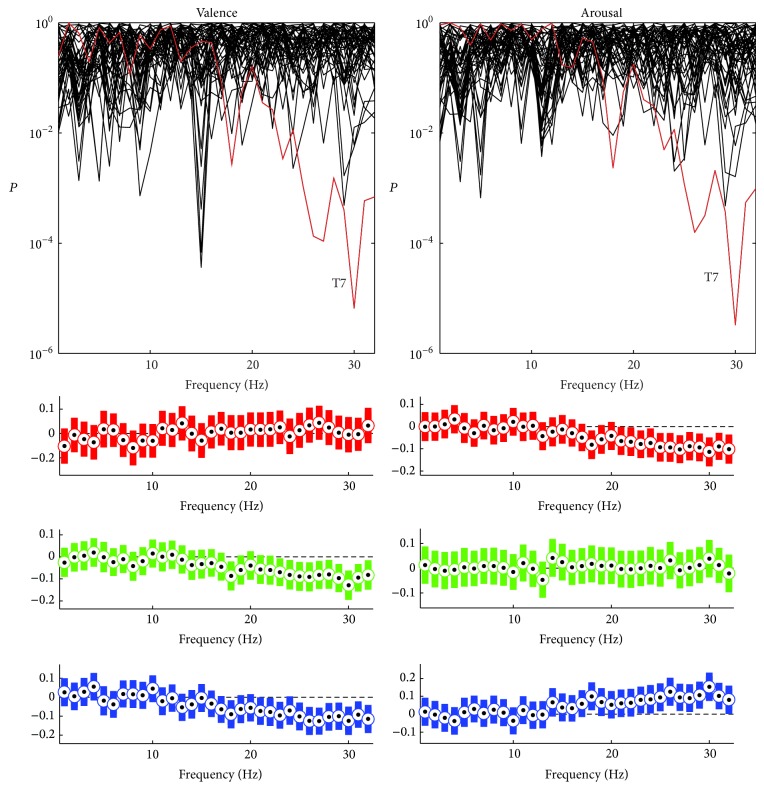
The results of the statistical analysis for valence and arousal. In the upper row, the *P* values for the one-way ANOVA test are given separately for all of the features representing the activity in single frequencies between 1 and 32 Hz. Each trend corresponds to either a single channel or a channel pair. T7 is highlighted with red color. Below are the results of Tukey post hoc honestly significant difference test carried out separately for the features representing the activity in single frequencies of channel T7. Bars indicate the estimated difference in the class means and the 95% confidence intervals. For valence the comparison is made between the classes “unpleasant” and “neutral valence” (red), “unpleasant” and “pleasant” (green), and “neutral valence” and “pleasant” (blue). For arousal the comparison is made between “calm” and “medium arousal” (red), “calm” and “excited/activated” (green), and “medium arousal” and “excited/activate” (blue). If the bar does not contain value 0, the class means differ statistically significantly (*P* < 0.05).

In Figure 3, the topographical distribution of features resulting in the best classification rate is presented for valence. The channel-wise contribution of different frequencies is given as well. Whereas the SPD features were practically absent in the set, the SP features could be seen to fall into three topographic clusters. This division was robustly seen also in different phases of feature selection presented in Figure 2. Most of the features were selected from the temporal area of the left hemisphere and represented high frequencies (15–32 Hz). These frequencies were also present in the second cluster, located in the parieto-occipital area of right hemisphere. Low frequencies (<6 Hz) were seen in all three topographic clusters being most prominent in the frontal area. The number of features might not, however, be an optimal measure for the importance of certain channels or frequencies.  Figure 4 addresses this problem by showing how the classification rate for valence was affected if the features representing certain channel were removed. As expected the results correlated well with those illustrated in Figure 3 indicating that the channels represented by many of the features were also the most important ones in terms of the classification.  Figure 4 also shows the channel-wise feature values for the three different classes of valence. In several channels of the left hemisphere's temporal and right hemisphere's parieto-occipital areas, increased high-frequency (15–32 Hz) activity was associated with “pleasant.” Most dramatically this was seen in T7, in which the feature values were found to be statistically significantly higher compared to those of “neutral valence” and “unpleasant” in almost all frequencies as illustrated in Figure 5. No significant difference was found between “unpleasant” and “neutral valence.” The “pleasant” was also associated with decreased low-frequency (<6 Hz) activity in all three above-mentioned topographic clusters.

### 3.2. Arousal

**Figure 6:**
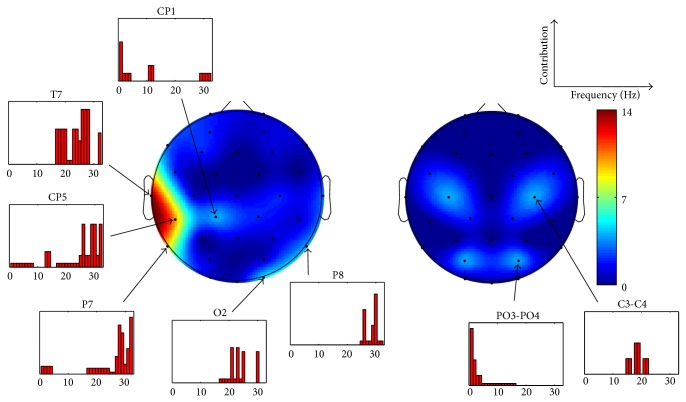
The topographical distribution of features and the channel-wise contribution of different frequencies in the best performing feature set for arousal. The feature set contained 90 features. Spectral power (on the left) and spectral power difference (on the right) features are illustrated separately. The topographic plots are given in absolute scale representing the number of features.

**Figure 7:**
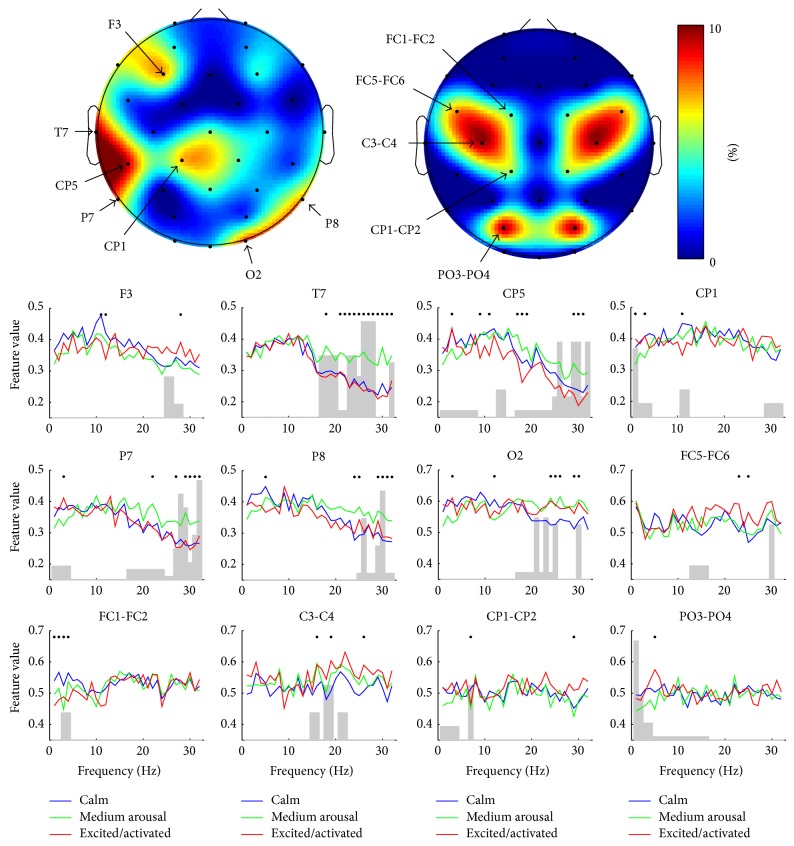
The importance of different channels and frequencies in the classification of arousal. The topographic plots illustrate channel-wise how the classification performance is decreased if all the features in that specific channel are excluded from the best performing feature set. The plots have been made separately for the spectral power (on the left) and the spectral power difference (on the right) features. The channel-wise feature values for the three different classes of arousal are given below the topographic plots. The curves are the mean values of the features representing the single frequencies. The dots above the curves indicate that the values between the classes in that specific frequency differ statistically significantly (*P* < 0.05) according to one-way ANOVA test. The gray bars represent the contribution of different frequencies in that specific channel in the best performing feature set.

Figure 6 presents a similar topographical distribution of features and channel-wise contribution of frequencies for arousal already illustrated for valence. The role of the left hemisphere's temporal lobe seemed to be even more dominant in the best performing feature set of arousal than it was for valence. This feature distribution was seen throughout the feature selection procedure (see Figure 2). Again, most of the features represented high frequencies (15–32 Hz) which were also apparent in the parieto-occipital area. The contribution of SPD features in the feature set was higher for arousal than it was for valence. The results are further analyzed in Figure 7, in which the importance of different channels for the classification performance as well as the channel-wise feature values for the three different classes of arousal are exposed. The most dominant finding was the association between the increased high-frequency (15–32 Hz) activity and “medium arousal.” This association was seen mostly in the left temporal area (see Figure 5 for the results of statistical analysis) but also partly in the right parieto-occipital area. It should be noted that, based on the nine emotional keywords used, the samples annotated as “medium arousal” were exactly the same annotated as “pleasant” representing the keywords amusement and joy/happiness. The figure also shows some associations between “calm” and decreased high-frequency (15–32 Hz) activity in O2 and C3-C4 leads, as well as increased low-frequency (<6 Hz) activity in FC1-FC2 lead.

### 3.3. Validation of the Results

**Figure 8:**
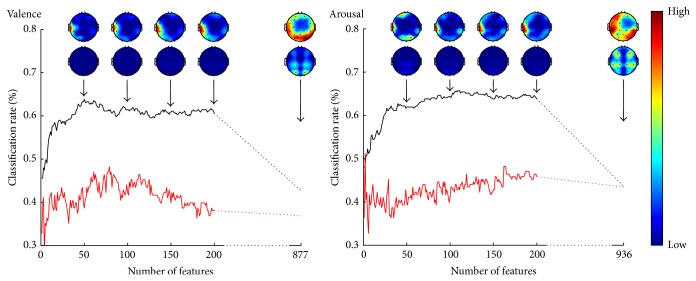
The classification performance for training (black curve) and testing (red curve) sets and the topographical distribution of features as a function of number of features for valence and arousal. The topographic plots are given in relative scale red indicating high and blue low number of features. Spectral power (above) and spectral power difference (below) features are illustrated separately for 50, 100, 150, 200, and all preselected features.

**Figure 9:**
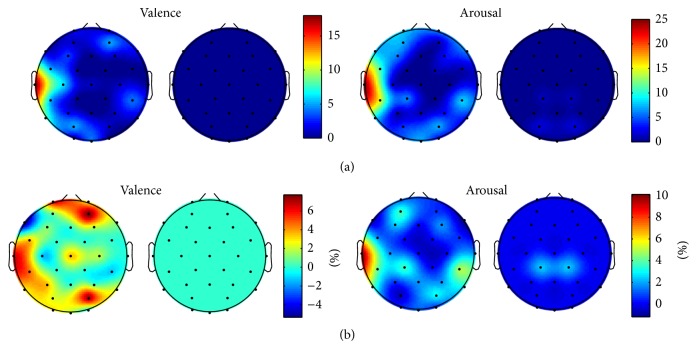
(a) The topographical distribution of features and (b) the importance of different channels in the classification of testing data for valence and arousal. The results are given for the feature sets that resulted in the best classification rate for the testing data containing 79 and 162 features for valence and arousal, respectively. The plots are in absolute scale representing the number of features (a) and the percentage how much the classification performance is decreased if all the features in that specific channel are excluded from the feature set (b). Spectral power (on the left column) and spectral power difference (on the right column) features are illustrated separately for both valence and arousal.

The results of the validation procedure carried out with the separate training and testing sets are illustrated in Figure 8. The classification performance for both sets as well as the topographical distribution of features in different phases of the feature selection is given. The topographical distribution of features resembled closely that of achieved with the whole dataset emphasizing the role of the left hemisphere's temporal area for both valence and arousal. As expected, dividing the data into two subsets affected the generalizability of the feature set optimized for training data leading to a fundamentally lower classification rate for testing data. The finding indicates the vulnerability of a rather small dataset with a high interindividual and intersample variation. The figure shows, however, similar behavior between the classification rates of training and testing sets suggesting that, with feature selection approach carried out, a significant overlearning is unlikely.  Figure 9 shows the topographical distribution of features and importance of different channels in the classification of testing data for valence and arousal. The figure verifies that the results achieved with independent training and testing sets were similar to those presented above for the whole dataset.

**Figure 10:**
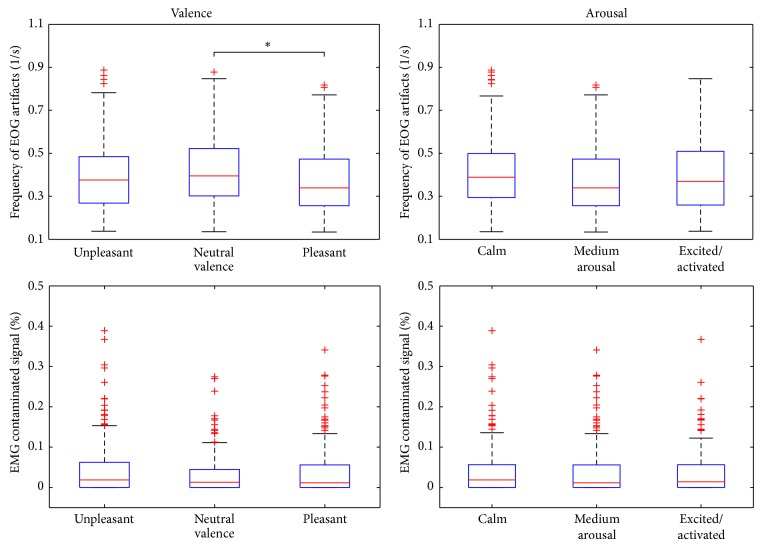
The contribution of EOG and EMG artifact to the signal for the three different classes of valence and arousal. The upper row represents the average of the frequency of EOG artifacts calculated from Fp1 and Fp2 leads. The lower row shows the percentage of EMG contaminated signal in T7. Statistical comparison between all three pairs of classes was performed with Mann-Whitney *U* test (^∗^ *P* < 0.05).

The contribution of EOG and EMG artifacts was estimated based on the findings and the results are illustrated in Figure 10. The difference between the frequency of EOG artifacts in the samples representing “neutral valence” and “pleasant” was found to be statistically significant suggesting that, while watching videos with pleasant content, the subjects move and blink their eyes less. As the power of EOG artifacts mainly lies in the lower frequencies, the phenomenon may have contributed to the above-resented findings related to the frontal area. The contribution of EMG artifact was found to be comparable in all three classes of valence and arousal. Transient EMG artifacts hence did not explain the reported difference in the high-frequency activity of the left temporal area.

## 4. Discussion

In this study, the EEG characteristics related to video-induced emotions were explored. The problem was approached from a classifier perspective: the features that performed best in classifying person's valence and arousal while watching audiovisual material with emotional content were searched. Compared to the previous studies, the analysis was not restricted to the classical frequency bands of EEG. Instead, the features optimizing the classification were selected from a large set created using a high number of partly overlapping frequency bands of varying width. The proposed classifier-based approach was able to reveal a clear association between the increased high-frequency (15–32 Hz) activity in the left temporal area and the samples classified as “pleasant” and “medium arousal.” These samples represented the emotional keywords amusement and joy/happiness. The finding suggests the occurrence of a specific neural activation during video-induced pleasant emotion and the possibility to detect this from the left temporal area using EEG. The results thus offer valuable new information from both neuroscience and technological point of view.

While the asymmetric cortical electrical activity related to emotional processing is well known, the previous studies have mainly focused on assessing the changes in the prefrontal area [27]. In the current study, left temporal area was found to be most informative when it comes to detecting pleasant affect. Several observations in the literature support the role of temporal cortex in the processing of audiovisual material with emotional content. The superior temporal regions are considered to be in a key role when it comes to the face perception [28, 29] and the recognition of facial expressions of emotions has been reported to induce even stronger activation of these areas than simple face detection [30]. While being responsible for the speech and linguistically relevant sound processing [31], the superior temporal cortex has also proved to be more responsive to the emotional tone of voice compared to that of neutral tone [32, 33]. Since both audio and visual emotional stimuli have been shown to activate superior temporal regions, a possible general role for the perception of emotional expressions has been suggested for these areas [34, 35]. In line with this are the findings of Schellberg et al. [36] who reported the fast beta EEG activity at temporal locations to be indicative of the emotional state of people watching emotionally engaging films. Unlike in the current study, they found the right temporal area to be more indicative of the person's positive and negative emotional states. Interestingly, emotional responses after intracerebral electrical stimulation of temporal lobe have also been reported [37]. Our results support the important role of high-frequency temporal activity in the EEG-based emotion classification in addition to, for example, the well-known asymmetric frontal activity. While no compelling evidence of EEG markers consistently seen across different studies is observed, one can assume experimental factors such as study design, induced emotional stimulation, and/or emotion categorization to substantially affect the results.

In the present study, left hemisphere's temporal activity was found to most distinguishably carry the information about the subject's emotional experience. Since the emotional stimulus was induced by showing videos, both audio and visual aspects should be taken into account when considering the neural mechanisms behind the finding. According to the traditional notion, language functions arise from the left temporal area, whereas the right side is more specialized for the processing of music [28, 38]. Recently, Schirmer et al. [39] confirmed the left-lateralization for speech but failed to reveal clear right hemisphere dominance for music. On the other hand, a near-infrared spectroscopic study showed that the left temporal area is significantly activated when looking at happy faces [40]. An integrative role in the processing of audiovisual information has also been suggested for the left hemisphere's superior temporal cortex [41]. Consequently, whether our observation about the significance of left temporal area in the classification of person's emotional experience arises solely from audio or visual stimulation or originates from the integration of the emotional information from these modalities is yet to be confirmed.

People tend to convey their emotional state through facial expressions, due to which, in the current experimental setup, the EEG recordings were potentially subject to muscular artifact correlating with the emotion experienced. The spectrum of contracting striated muscle, measured using surface EMG, is known to represent a band of 20–300 Hz [42] which entirely overlaps with the high-frequency neural activity [43]. Ruling out the contribution of EMG to the findings is thus extremely important when exploring the emotion-induced cortical activity. Several facts, however, suggest that our results do not originate from muscular activity. The careful inspection of the recordings from left temporal area showed that transient EMG artifact did not explain the increased high-frequency activity related to positive valence. In fact, the amount of artifact was slightly, albeit not significantly, higher in the samples representing the unpleasant emotion compared to those of pleasant emotion. The only possible source for the finding, other than cortical, would thus have been static long-lasting muscle activity practically impossible to differentiate from EEG. The origin of static muscular activity does not, however, fit with the fact that one would assume to see this kind of rigidity in the facial area related to unpleasant emotion rather than pleasant. The phenomenon would also be likely to occur bilaterally, while our results refer to solely left side activity.

In the current study, a two-dimensional bipolar circumplex model of valence and arousal was used to describe the subjects' emotions. In the literature, additional dimensions, such as potency and unpredictability, to the model have also often been proposed [7]. The most commonly suggested addition, that is, potency, is typically justified by the inability of the two-dimensional valence-arousal space to effectively differentiate between fear and anger [44]. However, there is no solid consensus as to which, if any, additional dimensions are needed. Furthermore, valence and arousal have widely been accepted to carry the most relevant information regarding the emotional experience (see, e.g., [45]), due to which the bipolar circumplex model was also utilized in this study.

Compared to the previous studies, our approach for feature selection provided an exceptional and unique possibility to observe without restriction the characteristics of the features essential for EEG-based recognition of emotional experience. Conventionally, the technological solutions for this task have been based on assessing the activity changes in the classical frequency bands (see, e.g., [14, 15, 22]). The computational demands as well as the historical reasons have generally led to the restriction of the feature set in terms of frequency space even though using higher number of narrower bands has been proposed to improve the detection of emotional responses from the signal [21]. Consequently, our intention was to not restrict the frequency characteristics of the features to the fixed bands, but instead to observe more generally how the essential information is divided in the frequency space. Similarly, we wanted to provide a possibility for the classifier to choose the most informative combination of SP and SPD features as well as the features from different topographic locations. Interestingly, the most informative part of the signal was found to be on 15–32 Hz frequency band which overlaps almost entirely with the beta activity.

The large feature set compared to the number of samples comes with a risk of overlearning, which should be taken into account when interpreting the results. From the over 800 features preselected for both valence and arousal most likely some performed well in classification only by chance. Therefore, one should not make conclusive statements based on single features, but instead look at the more general frequency and topographical characteristics of the best performing feature set. Taking this into account, only the most substantial finding in terms of number of features, classification performance, and statistical significance, that is, the left temporal activation during pleasant emotion was highlighted when reporting the results of the current study.

Several aspects of the study will require further exploration. The small size of the dataset did not allow us to examine the findings separately for right and left handed subjects which should be carried out in future. The differences between genders should be assessed as well. As the subject's emotional state may also alter substantially during a single video clip, an analysis of the dynamical EEG changes would be likely to reveal even more clearly the effects of emotional experience than the features calculated over the whole sample. Since the contribution of EMG to the main finding could not be totally ruled out, it would be preferable to confirm the phenomenon also with a different modality, such as fMRI, that is not sensitive to muscle artifact. In addition, different movie clips should be used as the results may be to some extent specific to the video material.
